# Supplementary material for: Identification of Four Mouse FcRn Splice Variants and FcRn-Specific Vesicles
Source: Cells. 2024 Mar 29;13(7):594. doi: 10.3390/cells13070594 (PMC11012118; doi:10.3390/cells13070594)

Identification of four mouse FcRn splice variants and FcRn-specific vesicle

George Haddad[1] and Judith Blaine[1*]

1 Division of Renal Disease and Hypertension, Department of Medicine, University of Colorado School of Medicine, Aurora, CO, USA.

Supplemental figures

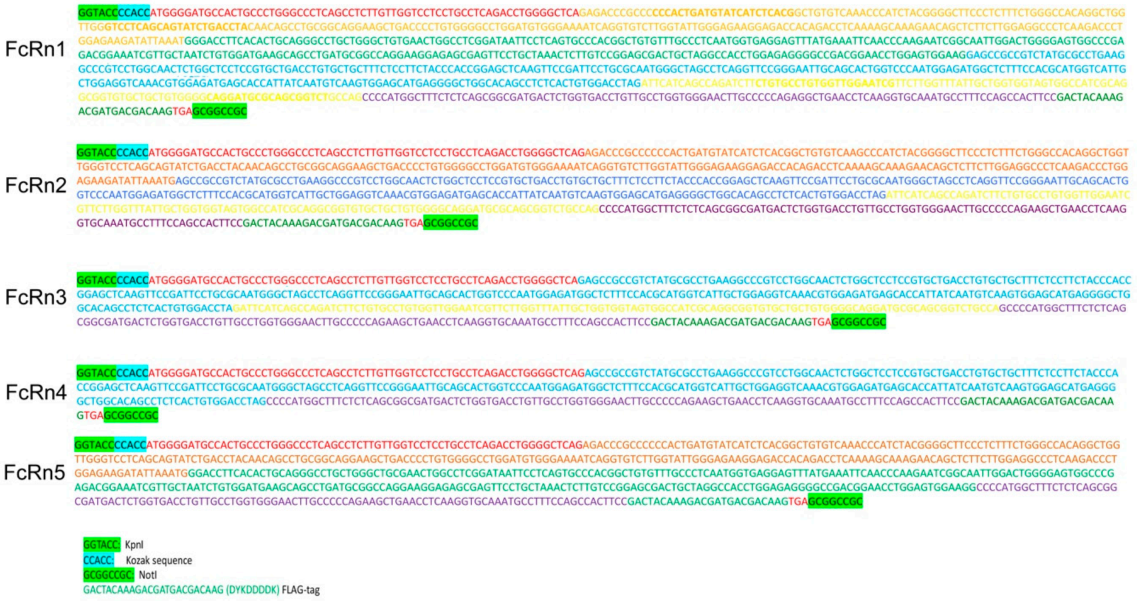

Suppl figure S1: The nucleotide sequences of the FcRn splice variants

The complete nucleotide sequences of each FcRn splice variant. The colors indicate the exons, red: exon 2, orange: exon 3, green: exon 4, blue: exon 5, yellow: exon 6, and purple: exon 7.

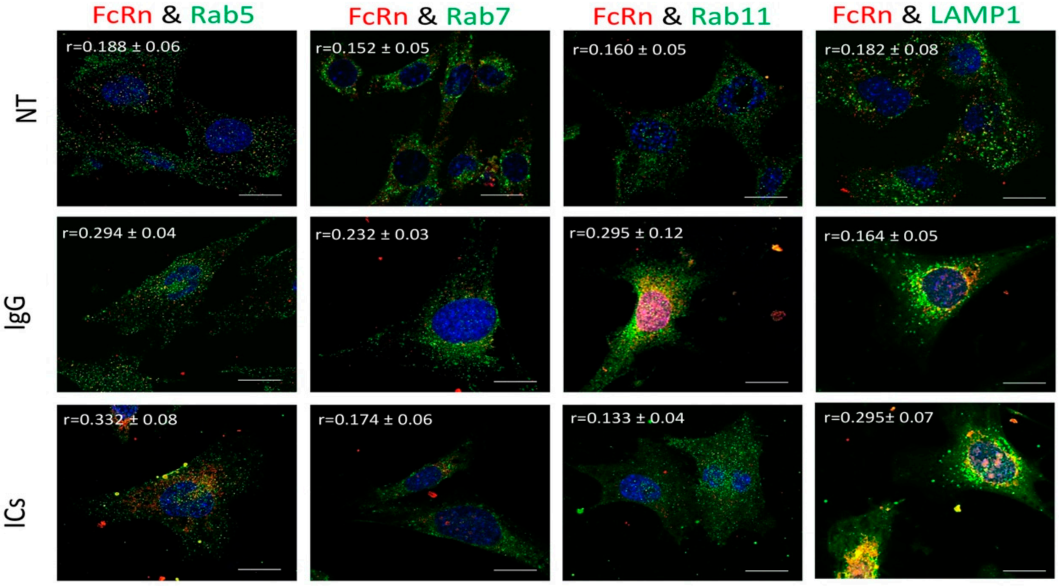

Suppl figure S2: Endogenous FcRn expression in wild-type mouse podocytes and its interaction with Rab5, 7, 11, and LAMP1 vesicles in the presence of monomeric IgG or immune complexes.

Monomeric IgG and immune complexes were added to the cells for 2 hours and incubated at 37 ºC and 5% CO_2. The cells were fixed and stained for Rab 5, 7, 11, and LAMP1 in addition to anti-mouse FcRn antibody and Hoechst 33342 nuclear stain. The images were obtained using Stedycon Abberior (STED) microscope and 100X objective lens. The scale bar represents 10 μm. The (r) values represent colocalization intensity plus/minus standard deviation.

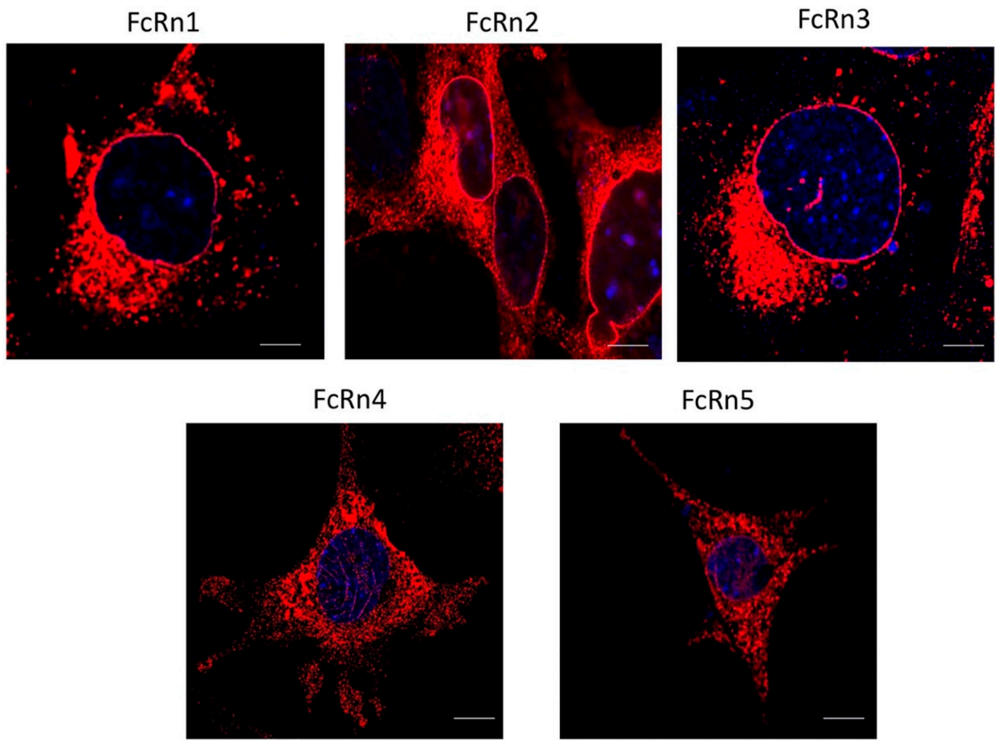

Suppl figure S3: FcRn variants expression in the nuclear envelope.

The FcRn-KO podocytes were infected with the lentivirus vectors of all the splice variants (FcRn1, 2, 3, 4, & 5) and the cells were imaged after 72 hours using an anti FLAG-tag antibody and Hoechst 33342 nuclear stain. The images were obtained using Stedycon Abberior (STED) microscope and 100X objective lens. The scale bar represents 10 μm.

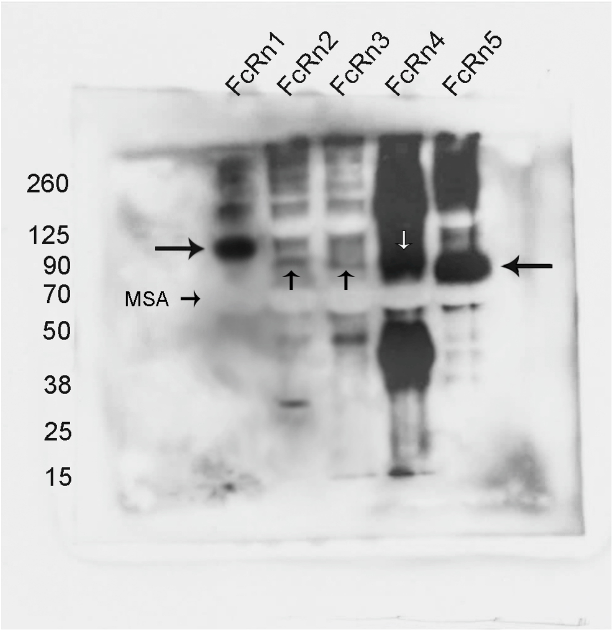

Suppl figure S4: FcRn splice variants interaction with mouse albumin

Mouse serum albumin (50 μg) was incubated with an equal concentration of purified recombinant FcRn1, 2, 3, 4 or 5 overnight at 37ºC in a PBS buffer pH 5.5. The solution was resolved using an SDS-PAGE gel and Western blot. The membrane was probed with an anti-FLAG tag antibody and an anti-species HRP-conjugated secondary antibody. Large arrows indicate the interaction of FcRn1 and 5 with albumin. The small arrows indicate the negative band of unbound albumin and interaction of FcRn2 and 3 with albumin. The white arrow indicates the interaction of FcRn4 and albumin.

hFcRn1

GGTACCCCACCATGGGGGTCCCGCGGCCTCAGCCCTGGGCGCTGGGGCTCCTGCTCTTTCTCCTTCCTGGGAGCCTGGGCGCAGAAAGCCACCTCTCCCTCCTGTACCACC
TTACCGCGGTGTCCTCGCCTGCCCCGGGGACTCCTGCCTTCTGGGTGTCCGGCTGGCTGGGCCCGCAGCAGTACCTGAGCTACAATAGCCTGCGGGGCGAGGCGGAGCC
CTGTGGAGCTTGGGTCTGGGAAAACCAGGTGTCCTGGTATTGGGAGAAAGAGACCACAGATCTGAGGATCAAGGAGAAGCTCTTTCTGGAAGCTTTCAAAGCTTTGGG
GGGAAAAGGTCCCTACACTCTGCAGGGCCTGCTGGGCTGTGAACTGGGCCCTGACAACACCTCGGTGCCCACCGCCAAGTTCGCCCTGAACGGCGAGGAGTTCATGAAT
TTCGACCTCAAGCAGGGCACCTGGGGTGGGGACTGGCCCGAGGCCCTGGCTATCAGTCAGCGGTGGCAGCAGCAGGACAAGGCGGCCAACAAGGAGCTCACCTTCCTG
CTATTCTCCTGCCCGCACCGCCTGCGGGAGCACCTGGAGAGGGGCCGCGGAAACCTGGAGTGGAAGGAGCCCCCCTCCATGCGCCTGAAGGCCCGACCCAGCAGCCCT
GGCTTTTCCGTGCTTACCTGCAGCGCCTTCTCCTTCTACCCTCCGGAGCTGCAACTTCGGTTCCTGCGGAATGGGCTGGCCGCTGGCACCGGCCAGGGTGACTTCGGCCCC
AACAGTGACGGATCCTTCCACGCCTCGTCGTCACTAACAGTCAAAAGTGGCGATGAGCACCACTACTGCTGCATTGTGCAGCACGCGGGGCTGGCGCAGCCCCTCAGGGT
GGAGCTGGAATCTCCAGCCAAGTCCTCCGTGCTCGTGGTGGGAATCGTCATCGGTGTCTTGCTACTCACGGCAGCGGCTGTAGGAGGAGCTCTGTTGTGGAGAAGGATG
AGGAGTGGGCTGCCAGCCCCTTGGATCTCCCTTCGTGGAGACGACACCGGGGTCCTCCTGCCCACCCCAGGGGAGGCCCAGGATGCTGATTTGAAGGATGTAAATGTGA
TTCCAGCCACCGCCTGAGCGGCCGC

hFcRn2

GGTACCCCACCATGGGGGTCCCGCGGCCTCAGCCCTGGGCGCTGGGGCTCCTGCTCTTTCTCCTTCCTGGGAGCCTGGGCGCAGGTCCCTACACTCTGCAGG
GCCTGCTGGGCTGTGAACTGGGCCCTGACAACACCTCGGTGCCCACCGCCAAGTTCGCCCTGAACGGCGAGGAGTTCATGAATTTCGACCTCAAGCAGGGC
ACCTGGGGTGGGGACTGGCCCGAGGCCCTGGCTATCAGTCAGCGGTGGCAGCAGCAGGACAAGGCGGCCAACAAGGAGCTCACCTTCCTGCTATTCTCCTG
CCCGCACCGCCTGCGGGAGCACCTGGAGAGGGGCCGCGGAAACCTGGAGTGGAAGGAGCCCCCCTCCATGCGCCTGAAGGCCCGACCCAGCAGCCCTGG
CTTTTCCGTGCTTACCTGCAGCGCCTTCTCCTTCTACCCTCCGGAGCTGCAACTTCGGTTCCTGCGGAATGGGCTGGCCGCTGGCACCGGCCAGGGTGACTTC
GGCCCCAACAGTGACGGATCCTTCCACGCCTCGTCGTCACTAACAGTCAAAAGTGGCGATGAGCACCACTACTGCTGCATTGTGCAGCACGCGGGGCTGGCG
CAGCCCCTCAGGGTGGAGCTGGCCCCTTGGATCTCCCTTCGTGGAGACGACACCGGGGTCCTCCTGCCCACCCCAGGGGAGGCCCAGGATGCTGATTTGAA
GGATGTAAATGTGATTCCAGCCACCGCCTGAGCGGCCGC

hFcRn3

GGTACCCCACCATGGGGGTCCCGCGGCCTCAGCCCTGGGCGCTGGGGCTCCTGCTCTTTCTCCTTCCTGGGAGCCTGGGCGCAGAGCCCCCCTCCATGCGC
CTGAAGGCCCGACCCAGCAGCCCTGGCTTTTCCGTGCTTACCTGCAGCGCCTTCTCCTTCTACCCTCCGGAGCTGCAACTTCGGTTCCTGCGGAATGGGCTG
GCCGCTGGCACCGGCCAGGGTGACTTCGGCCCCAACAGTGACGGATCCTTCCACGCCTCGTCGTCACTAGCAGTCAAAAGTGGCGATGAGCACCACTACTG
CTGCATTGTGCAGCACGCGGGGCTGGCGCAGCCCCTCAGGGTGGAGCTGGAATCTCCAGCCAAGTCCTCCGTGCTCGTGGTGGGAATCGTCATCGGTGTC
TTGCTACTCACGGCAGCGGCTGTAGGAGGAGCTCTGTTGTGGAGAAGGATGAGGAGTGGGCTGCCAGCCCCTTGGATCTCCCTTCGTGGAGACGACACCG
GGGTCCTCCTGCCCACCCCAGGGGAGGCCCAGGATGCTGATTTGAAGGATGTAAATGTGATTCCAGCCACCGCCTGAGCGGCCGC

hFcRn4

GGTACCCCACCATGGGGGTCCCGCGGCCTCAGCCCTGGGCGCTGGGGCTCCTGCTCTTTCTCCTTCCTGGGAGCCTGGGCGCAGAGCCCCCCTCCATGCG
CCTGAAGGCCCGACCCAGCAGCCCTGGCTTTTCCGTGCTTACCTGCAGCGCCTTCTCCTTCTACCCTCCGGAGCTGCAACTTCGGTTCCTGCGGAATGGGC
TGGCCGCTGGCACCGGCCAGGGTGACTTCGGCCCCAACAGTGACGGATCCTTCCACGCCTCGTCGTCACTAACAGTCAAAAGTGGCGATGAGCACCACTA
CTGCTGCATTGTGCAGCACGCGGGGCTGGCGCAGCCCCTCAGGGTGGAGCTGGCCCCTTGGATCTCCCTTCGTGGAGACGACACCGGGGTCCTCCTGCCC
ACCCCAGGGGAGGCCCAGGATGCTGATTTGAAGGATGTAAATGTGATTCCAGCCACCGCCTGAGCGGCCGC

hFcRn5

GGTACCCCACCATGGGGGTCCCGCGGCCTCAGCCCTGGGCGCTGGGGCTCCTGCTCTTTCTCCTTCCTGGGAGCCTGGGCGCAGAATCTCCAGCCAAGTCC
TCCGTGCTCGTGGTGGGAATCGTCATCGGTGTCTTGCTACTCACGGCAGCGGCTGTAGGAGGAGCTCTGTTGTGGAGAAGGATGAGGAGTGGGCTGCCAG
CCCCTTGGATCTCCCTTCGTGGAGACGACACCGGGGTCCTCCTGCCCACCCCAGGGGAGGCCCAGGATGCTGATTTGAAGGATGTAAATGTGATTCCAGCC
ACCGCCTGAGCGGCCGC

hFcRn6

GGTACCCCACCATGGGGGTCCCGCGGCCTCAGCCCTGGGCGCTGGGGCTCCTGCTCTTTCTCCTTCCTGGGAGCCTGGGCGCAGCCCCTTGGATCTC
CCTTCGTGGAGACGACACCGGGGTCCTCCTGCCCACCCCAGGGGAGGCCCAGGACGCTGATTTGAAGGATGTAAATGTGATTCCAGCACCGCCTGA
GCGGCCGC

GGTACC KpnI
CCACC Kozak sequence
GCGGCCGC NotI

Suppl figure S5: Human FcRn splice variants nucleotide sequences.

The complete nucleotide sequences of each human FcRn splice variant. The colors indicate the exons, red: exon 4 part1, dark red: exon 4 part 2, orange: exon 4 part 3, light green: exon 9 part 1, dark green: exon 9 part 2, and blue: exon 10. The genetic representation was modeled based on Ensembl.org accession # ENSG00000104870 FCGRT gene description.

Suppl Table S1: Mass spectrometry results of intracellular vesicles specific protein detection in FcRn splice variants

Protein	**FcRn1**	**FcRn2**	**FcRn3**	**FCRn4**	**FcRn5**	**endo FcRn**
**Rab1A**	526,574	596,262	160,053	272,383	178,168	108,444
**Rab1B**	59,613	111,496	18,557	37,507	18,052	10,124
**Rab2A**	174,378	246,100	87,375	62,469	74,502	9,879
**Rab4B**	43,472	85,491	14,133	12,091	0	0
**Rab5A**	44,576	51,634	21,849	31,729	0	21,705
**Rab5B**	46,991	61,647	20,590	23,152	19,828	18,897
**Rab5C**	312,885	344,955	77,714	182,524	189,600	78,035
**Rab6A**	432,818	487,236	193,314	235,751	222,238	17,330
**Rab7A**	778,746	1,093,520	410,256	435,443	537,827	165,500
**Rab8A**	14,018	37,638	19,509	12,526	9,794	7,540
**Rab8B**	10,063	10,574	0	0	0	0
**Rab10**	30,980	64,503	22,826	14,525	14,241	9,965
**Rab11B**	618,762	847,117	245,575	370,337	374,468	109,053
**Rab12**	0	23,444	0	0	0	0
**Rab13**	16,053	12,345	26,366	9,764	14,623	8,844
**Rab14**	762,135	822,290	201,085	404,880	280,809	146,979
**Rab18**	124,997	180,783	78,611	56,114	18,626	0
**Rab21**	40,972	56,332	11,354	15,383	0	10,106
**Clathrin**	187,532	210,571	238,292	114,564	42,537	102,128

Uncropped Western Blots

Figure 1 blots

FcRn1

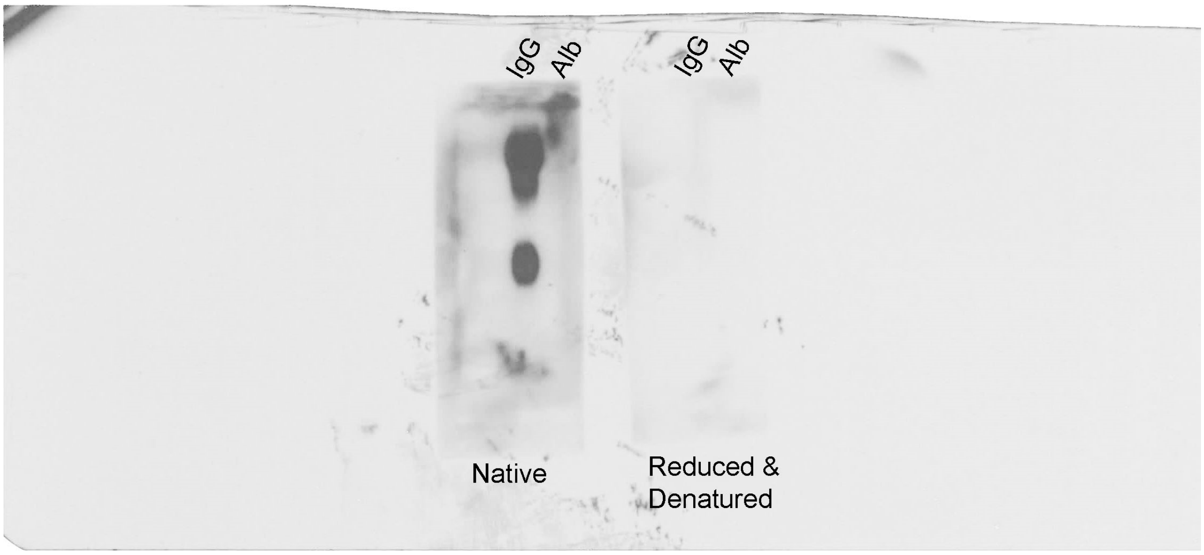

FcRn2

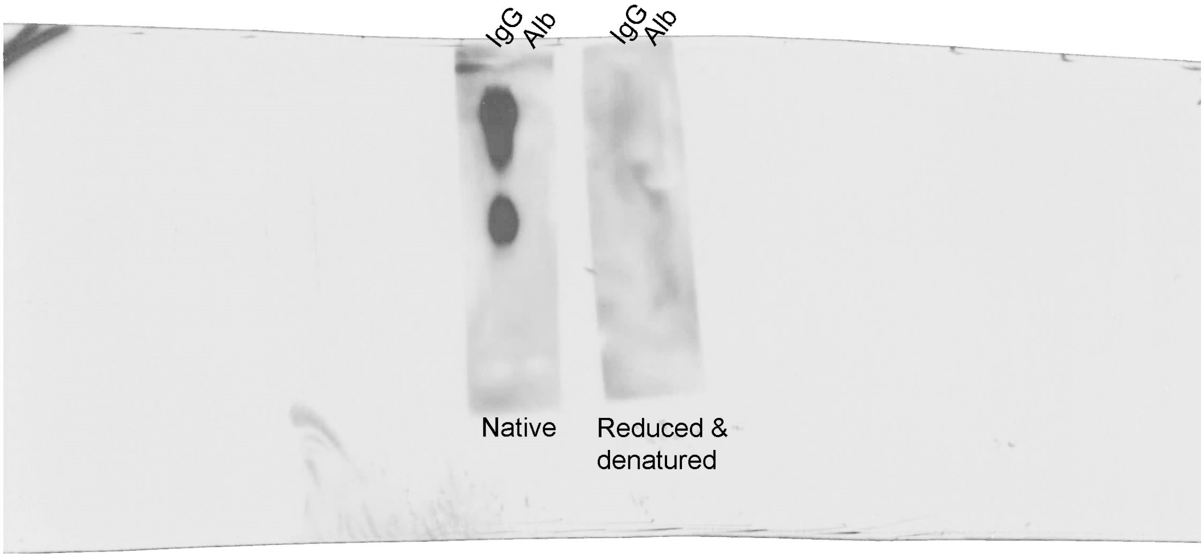

FcRn3

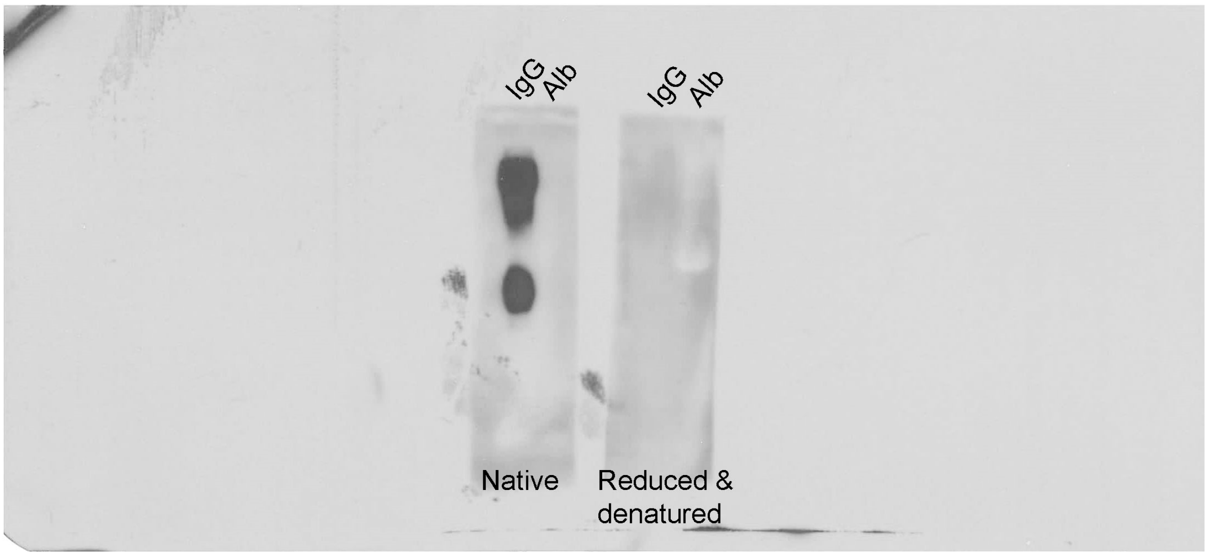
IgG
Alb
IgG
Alb
Native
Reduced &
denatured

FcRn4

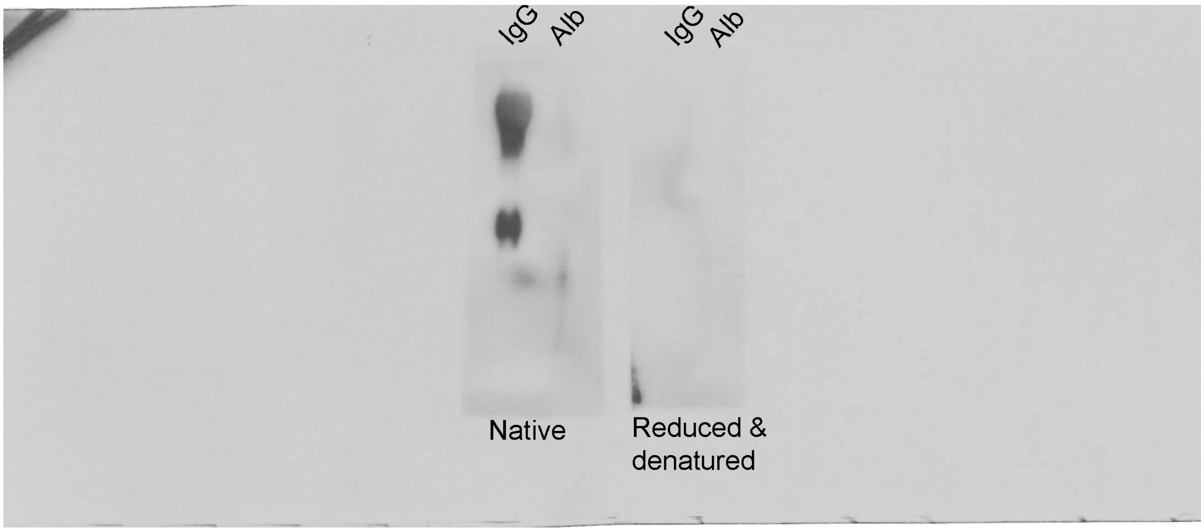
IgG
Alb
IgG
Alb
Native
Reduced &
denatured

FcRn5

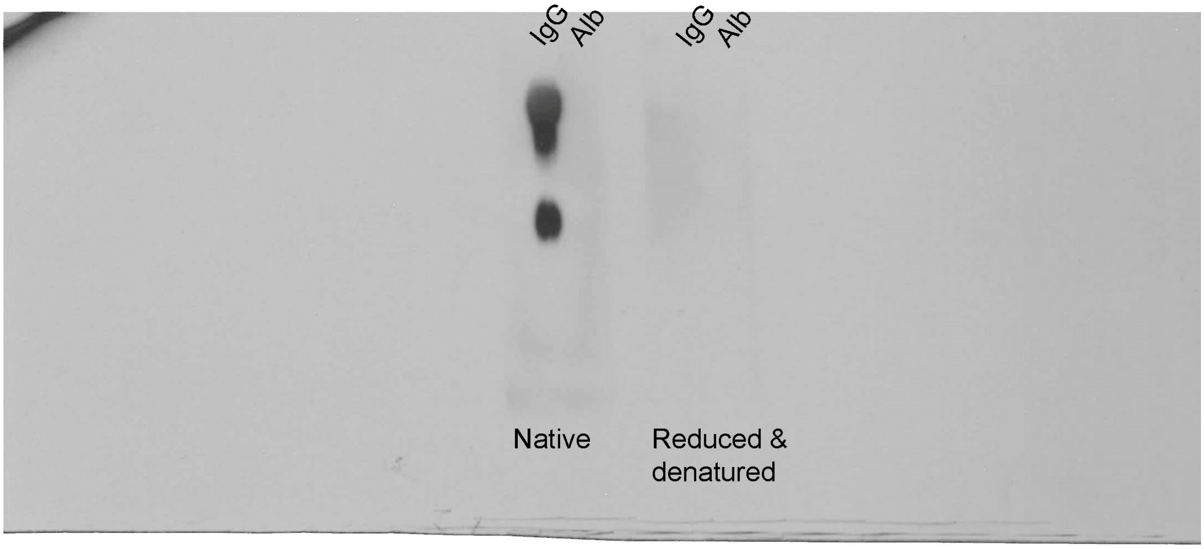

Supplement: Supplementary file 1 [file cells-13-00594-s001.zip › cells-2930259-supplementary.pdf]
